# Highly Photoluminescent and Stable N-Doped Carbon Dots as Nanoprobes for Hg^2+^ Detection

**DOI:** 10.3390/nano8110900

**Published:** 2018-11-02

**Authors:** Longshi Rao, Yong Tang, Hanguang Lu, Shudong Yu, Xinrui Ding, Ke Xu, Zongtao Li, Jin Z. Zhang

**Affiliations:** 1 Engineering Research Centre of Green Manufacturing for Energy-Saving and New-Energy Technology, School of Mechanical and Automotive Engineering, South China University of Technology, Guangzhou 510640, China; memerls@mail.scut.edu.cn (L.R.); ytang@scut.edu.cn (Y.T.); guyuexuan1999@foxmail.com (H.L.); shudong.yu@partner.kit.edu (S.Y.); dingxr@scut.edu.cn (X.D.); 2 Department of Chemistry and Biochemistry, University of California, Santa Cruz, CA 95064, USA; kxu26@ucsc.edu; 3 Light Technology Institute, Karlsruhe Institute of Technology (KIT), Engesserstrasse 13, 76131 Karlsruhe, Germany; 4 Department of Chemistry and Chemical Engineering, Chongqing University, Chongqing 400044, China

**Keywords:** microreactor, carbon dots, porous copper fibers, Hg^2+^ detection

## Abstract

We developed a microreactor with porous copper fibers for synthesizing nitrogen-doped carbon dots (N-CDs) with a high stability and photoluminescence (PL) quantum yield (QY). By optimizing synthesis conditions, including the reaction temperature, flow rate, ethylenediamine dosage, and porosity of copper fibers, the N-CDs with a high PL QY of 73% were achieved. The PL QY of N-CDs was two times higher with copper fibers than without. The interrelations between the copper fibers with different porosities and the N-CDs were investigated using X-ray photoelectron spectroscopy (XPS) and Fourier Transform infrared spectroscopy (FTIR). The results demonstrate that the elemental contents and surface functional groups of N-CDs are significantly influenced by the porosity of copper fibers. The N-CDs can be used to effectively and selectively detect Hg^2+^ ions with a good linear response in the 0~50 μM Hg^2+^ ions concentration range, and the lowest limit of detection (LOD) is 2.54 nM, suggesting that the N-CDs have great potential for applications in the fields of environmental and hazard detection. Further studies reveal that the different *d* orbital energy levels of Hg^2+^ compared to those of other metal ions can affect the efficiency of electron transfer and thereby result in their different response in fluorescence quenching towards N-CDs.

## 1. Introduction

Water pollution by heavy metal ions due to the emission of industrial waste presents a major environmental problem [1,2,3]. In particular, the mercury ion (Hg^2+^) is one of the most prevalent and dangerous pollutants, with the character of easy bioaccumulation and high toxicity, which causes serious health and environment problems, including damage to the nervous system, ecosystem, brain, and heart [4,5,6,7]. Furthermore, the water soluble Hg^2+^ is one of the most common forms in mercury pollution, which is hard to remove once released into the environment or getting into the human body due to its non-biodegradability [8,9,10]. As a consequence, sensitive and selective detection of Hg^2+^ is highly desired.

To date, several methods, such as anodic stripping voltammetry [11], Auger-electron spectroscopy [12], inductively coupled plasma mass spectrometry (IC-PMS) [13], atomic absorption spectrometry (AAS) [14], atomic fluorescence spectroscopy (AFS) [15], and X-ray absorption spectroscopy (XBS) [16] have been applied for the detection of Hg^2+^ ions. Nevertheless, these techniques need large-scale instruments, complex sample preparation, time-consuming procedures, and trained personnel, which restrict their practical applications in the routine detecting of Hg^2+^ [17,18]. Compared to these conventional methods, the fluorescent probe method has drawn considerable attention due to its advantages of including an easy operation, being nondestructive, having a high sensitivity, and exhibiting a rapid response for Hg^2+^ detection [19,20,21]. At present, many fluorescent probes based on quantum dots, organic fluorescent materials, and noble-metal nanoclusters have been developed [22,23,24]. However, their practical applications have been limited due to their disadvantages, including the toxic materials involved, low photostability, low selectivity, and complex preparation process. Thus, in order to improve the detection performance for practical use, new fluorescent probes need to be developed.

Currently, carbon dots (CDs) as fluorescent nanoprobes have attracted considerable attention because of their prominent water solubility, hypotoxicity, high biocompatibility, and excellent stability [25,26,27]. All of these novel properties set them apart from conventional fluorophores and identify them as promising a prospect for applications in catalysis [28], biosensing [29], medical diagnostics [30], the detection of metal ions [31], and photovoltaic devices [32]. However, the relatively low photoluminescence (PL) quantum yield (QY) and reproductivity of CDs greatly restrict their practical applications. To address these issues, much work has been performed to enhance the synthesis and properties of CDs, including doping with heteroatoms (such as N, P, S, Se, and Bi) as a facile method to tune their optical properties. For example, Dong et al. [33] reported N and S co-doped CDs prepared from L-cysteine and citric acid, with a PL QY of up to 73%. Zhang and co-workers [34] synthesized nitrogen-doped CDs (N-CDs) with strong blue luminescence, achieving a PL QY of 35.4%. Xavier’s group [35] developed an environmentally friendly and high efficient fluorescence sensor based on N-CDs. The as-prepared N-CDs with a PL QY of 34.5% showed an excellent selectivity towards Hg^2+^ ions. In addition, Venkateswarlu et al. [36] reported a solvent-free method to synthesize CDs from mushrooms. The resulting CDs exhibit a good stability with a high PL QY of up to 25% and can be effectively used to detect Hg^2+^ ions. However, the large-scale and rapid synthesis of particular CDs with desired luminescence properties is still very challenging.

In this study, highly photoluminescent N-CDs were synthesized using a microreactor with different porosities of copper fibers. By optimizing the synthesis conditions, the N-CDs were prepared on a large scale with a high PL QY of up to 73%. In addition, we compared the PL QY of N-CDs with and without copper fibers, and with or without structures. The relationship between copper fibers with different porosities and the N-CDs was studied by a combination of spectroscopy and microscopy techniques, revealing that the elemental contents and surface functional groups of N-CDs are greatly influenced by the porosity of the copper fibers. The as-prepared N-CDs were completely water-soluble and remarkably stable under UV light illumination, ionic strengths, and ambient air. The N-CDs were further applied to detect Hg^2+^ ions, achieving the LOD of 2.54 nM, showing their promising prospect of bioscience and toxic or hazardous materials detection. Moreover, the possible mechanism of fluorescence quenching of the N-CDs by Hg^2+^ was investigated by fluorescence lifetime and cyclic voltammetry studies.

## 2. Materials and Methods

### 2.1. Chemicals and Materials

Ethylenediamine (EDA), citric acid (CA), quinine sulfate (QS), tetrabuty-lammonium hexafluorophosphate (Bu_4_NBF_6_), dimethylformamide (DMF), sodium chloride (NaCl), manganese dioxide (MnCl_2_), calcium chloride (CaCl_2_), cadmium chloride (CdCl_2_), zinc chloride (ZnCl_2_), magnesium chloride (MgCl_2_), chromium chloride (CrCl_3_), mercury chloride (HgCl_2_), nickel chloride (NiCl_2_), ferrous chloride (FeCl_2_), barium chloride (BaCl_2_), lead chloride (PbCl_2_), copper chloride (CuCl_2_), hydrochloric acid (HCl), and sodium hydroxide (NaOH) were purchased from Shanghai Aladdin Biochemical Technology Co. LTD (Shanghai, China). Distilled water purchased from Guangzhou Qian Hui Chemical Glass Instrument Co. LTD (Guangzhou, China) was used as the water source.

### 2.2. Synthesis of N-CDs

**Scheme 1:**
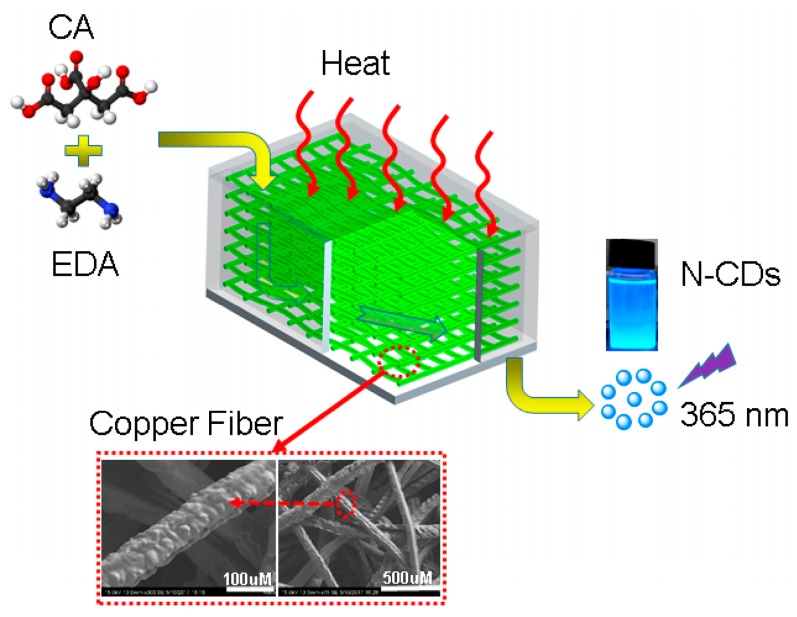
Illustration of the synthesis of N-CDs using a microreactor with porous copper fibers. CA and EDA are citric acid and EDA, respectively. The bottom SEM images are for the copper fibers used in the microreactor.

Scheme 1 shows a schematic illustration of the preparation process of N-CDs using a microreactor with porous copper fibers. The most important reaction area is the copper fibers (green region) with different porosities, which allows the transition from a liquid state to a gaseous state. The surface morphologies of copper fibers with 98%, 95%, 90%, 85%, and 80% porosities were performed by scanning election microscopy (SEM), as shown in the bottom of Scheme 1. For the synthesis of N-CDs, EDA (1.0 mL) and CA (1.5 g) were slowly added to 40 mL deionized water. After that, the precursors were injected by a fluid pump via a steel tube (inner diameter = 1.5 mm) into the microreactor. The temperatures of the microreactor were controlled by a digital temperature control instrument. It is worth noting that a one-way valve was used to block any liquid or vapour refluxing. The as-prepared products were continuously gathered by a three-necked flask. In addition, a UV analyzer was put under the three-necked flask to observe the products in site. The samples were further dialyzed (1000 Da) to remove the residual reagents. The final samples were collected through freeze drying and stored in a drying cabinet for further use.

### 2.3. Detection of Hg^2+^ Ions

To test the feasibility of the N-CDs to detect Hg^2+^, various concentrations (from 0 to 1000 μM) of Hg^2+^ ions were prepared, and 0.20 mL of a particular concentration of Hg^2+^ ions was injected into 2.0 mL of N-CDs aqueous solution. The PL spectrum of the sample solution was measured with 365 nm excitation. Meanwhile, the PL emission intensity of the N-CDs without and with Hg^2+^ ions was defined as I_0_ and I, respectively. In addition, to verity the selectivity of Hg^2+^, a series of metal ions, involving Fe^2+^, Ba^2+^, Hg^2+^, Zn^2+^, Cd^2+^, Pb^2+^, Na^+^, Mg^2+^, Ca^2+^, Cr^3+^, Mn^2+^, Ni^2+^, and Cu^2+^, were applied to detect the change of the N-CDs PL intensity. A solution of the above mentioned ions was prepared with a concentration of 0.50 mM and pH of 7.0. Afterwards, 0.20 mL of metal ions solution was loaded into 2.0 mL of N-CDs with intense stirring to achieve a homogeneous solution.

### 2.4. Characterizations

The morphology of porous copper fibers was determined using SEM (Merlin, Germany). The crystal structure of the N-CDs was characterized using an X-ray diffractometer (XRD, D8-Advance, Bruker, Karlsruhe, Germany) with a Cu-Kα radiation source (λ = 0.1542 nm) and a scanning angle (2θ) ranging from 5° to 80°. The crystal structure and surface morphology of the N-CDs were measured by a transmission electron microscope 2100F (TEM, JEOL, Tokyo, Japan) with an accelerating voltage of 200 kV. The XPS data were collected with an X-ray photoelectron spectrometer (Kratos Axis Ulra DLD, Kratos, Manchester, UK) with a mono Al-Kα excitation source (1486.6 eV). FTIR spectra were obtained from 4000 to 400 cm^−1^ on an FTIR spectrometer (Vertex 33, Bruker, Karlsruhe, Germany) in KBr discs after being vacuum-dried for 12 h. The UV-Vis absorption spectra of the N-CDs were achieved via a UV-Vis spectrometer (Shimadzu, Kyoto, Japan) over the wavelength range from 300 nm to 700 nm (1 nm interval). Cyclic voltammetry was carried out on a Solartron 1280B electrochemical workstation in a solution of Bu_4_NBF_6_ (0.10 M) in DMF at a scanning rate of 10 mV/s. A Pt sheet, a Pt wire, and an Ag/AgCl were used as the working electrode, the counter electrode, and the reference electrode, respectively. The PL spectrum of samples was recorded using a fluorescence spectrophotometer (RF-6000, Shimadzu). The PL QY of the N-CDs was calculated using the following equation [5,37]:

(1) $$\Phi =\Phi_{st}\;\frac{I}{I_{st}}\cdot \frac{A}{A_{st}}\cdot \frac{n^{2}}{n_{st}^{2}}$$

where the Φ is the PL QY, the subscript “*st*” denotes the reference quinine sulfate, “*I*” is the integrated fluorescence intensity, “*A*” is the UV-Vis absorption intensity at 365 nm, “*n*” is the different refractive index of the solvent, and the superscript “2” means the square of “*n*”. QS (PL QY is 0.54 at 365 nm) was used as the standard.

## 3. Results and Discussion

### 3.1. Structural and Optical Properties of N-CDs

**Figure 1:**
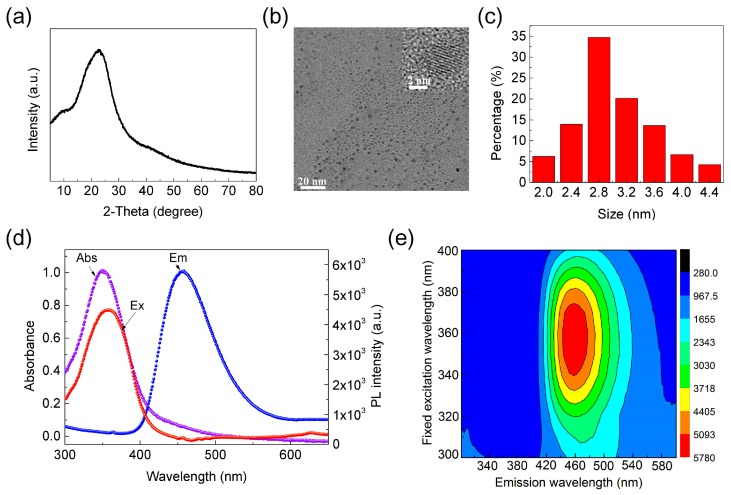
(**a**) XRD diffraction pattern of N-CDs. (**b**) TEM image of N-CDs. Inset is an HRTEM image exhibiting the well-separated, nearly monodispersed N-CDs. (**c**) The size distribution histograms of N-CDs, showing the average diameter of 2.8 ± 0.2 nm. (**d**) UV-Vis absorption (Abs, purple line), PL emission spectrum (Em, blue line), and PL excitation (Ex or PLE, red line) spectrum of the N-CDs. (**e**) Contour of the excitation spectra and emission spectra of the N-CDs.

XRD was used to characterize the crystal structure of the samples, as demonstrated in Figure 1a. The XRD pattern of the N-CDs has a single wide diffraction peak at 23.59°, showing a distorted graphitic structure with a small size. The TEM image in Figure 1b reveals that these N-CDs are uniform and well-dispersive. Moreover, the high-resolution TEM (HRTEM) image demonstrates remarkable crystalline characteristics, with a lattice distance of ~0.257 nm, corresponding to the (102) lattice planes of graphitic carbon. In addition, the size distribution histograms of the N-CDs (Figure 1c) exhibit a narrow size distribution and the average diameter is 2.8 ± 0.2 nm. In addition, the UV-Vis absorption spectra and the PL emission and excitation spectra of the N-CDs are displayed in Figure 1d. The synthesized N-CDs have an intense absorption band centered at 350 nm with a weak absorption in the visible region. The first characteristic absorption peak at 350 nm was possibly related to the absorption of an n-π^*^ transition or other functional groups of the N-CDs [38]. The PL emission spectrum has a strong peak at 460 nm, and the PL excitation (PLE) has a band that peaked at 365 nm when 460 nm PL was detected. The PLE peak is close to the 350 nm peak observed in the UV-Vis absorption spectrum, suggesting that the same species is responsible for both the absorption and PL in the N-CDs [39]. Photographs of the solution and powder of N-CDs are shown in Figure S1, suggesting the N-CDs with high PL QY can be achieved on a large scale. Furthermore, the contour plot (Figure 1e) of the PL/PLE spectra of N-CDs shows the strongest emission centered at about 460 nm when excited at the excitation wavelengths of 300–400 nm. The observed contour shows that the N-CDs have an excitation-independent emission behavior, indicating N-CDs with a narrow size distribution and a highly homogeneous surface structure, consistent with the TEM results.

### 3.2. Optimization of Synthesis Conditions

**Figure 2:**
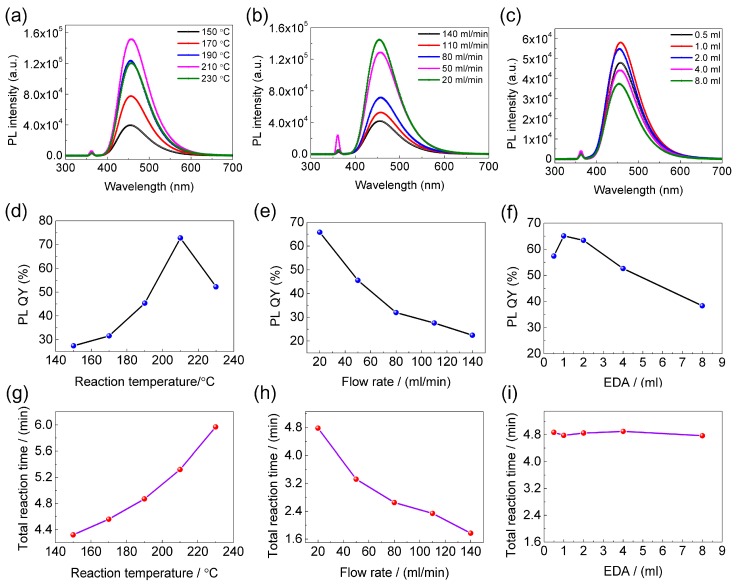
(**a**,**d**,**g**) The PL emission spectra, PL QY, and total reaction time of N-CDs synthesized at different reaction temperatures (150–230 °C), respectively. (**b**,**e**,**h**) The PL emission spectra, PL QY, and total reaction time of N-CDs synthesized at different flow rates (20–140 mL/min), respectively. (**c**,**f**,**i**) The PL emission spectra, PL QY, and total reaction time of N-CDs synthesized at different EDA dosages (0.5–8.0 mL), respectively. The porosity of copper fibers is 98%.

The PL QY of the N-CDs was significantly influenced by the reaction temperature, flow rate, EDA dosage, and porosity of copper fibers. Thus, it is necessary to optimize all the above synthesis conditions to obtain N-CDs with desired optical properties. Initially, the reaction temperature ranging from 150 °C to 230 °C at a 20 °C interval was investigated, as shown in Figure 2a,d,g. The flow rate is 20 mL/min, the dosage of EDA is 1.0 mL, and the porosity of copper fibers is 98%, and all samples maintain the same absorbance at 365 nm. Figure 2a demonstrates the PL spectra of the N-CDs that were synthesized at different reaction temperatures. When the temperature ranges from 150 °C to 230 °C, the PL intensity first increases and then decreases, reaching a maximum at 210 °C. PL QY of the N-CDs is calculated by using Equation (1) and QS as the standard (PL QY, 54%). As shown in Figure 2d, the PL QY increases gradually to a maximum value at a temperature of 210 °C, followed by a decrease at higher temperatures. The reaction is sufficient to improve the PL QY when the temperature increases. However, even higher temperatures (>210 °C) cause serious carbonization and reduce the PL QY. Besides, the N-CDs can be rapidly (the total reaction time is <6 min, showing in Figure 2g) and continuously synthesized, which shows a promising prospect for some applications, especially in hazardous materials detection and bioanalysis.

Moreover, the effect of the mixture flow rate on the optical properties of N-CDs was investigated, keeping other synthesis conditions the same, as shown in Figure 2b,e,h. As the flow rate increased, the PL intensity, PL QY, and total reaction time decreased. The flow rate influences the reaction time in the channel, and thus the nucleation and growth process of N-CDs. With a faster flow rate, the crystal carbon core grows inadequately, leading to a lower PL intensity and PL QY. In addition, the effects of the EDA dosage (Figure 2c,f,i) on the optical properties of N-CDs were further studied at a constant flow rate, reaction temperature, and porosity of copper fibers. Interestingly, the variations in PL intensity and PL QY are similar to Figure 2a,d, respectively, in that as the EDA dosage increases, both PL intensity and PL QY first rise and then fall. The optimum ratio of EDA dosage was 1.0 mL, displaying a PL QY of 65% (Figure 2f). When the EDA dosage was higher than 1.0 mL, the PL QY of the N-CDs was decreased. The epoxy groups and carboxyl groups on the surface of N-CDs are the centers of non-radiative recombination, and EDA can react with them to form amides and alcohols, which reduces the non-radiative recombination and enhances the PL emission of N-CDs. However, excessive alcohols will oxidize some of the carbon atoms of the N-CDs and increase the number of non-radiative recombination centers, resulting in a decrease of PL QY. Therefore, the optimized EDA dosage reduces N-CD surface defects and enhances PL QY.

**Figure 3:**
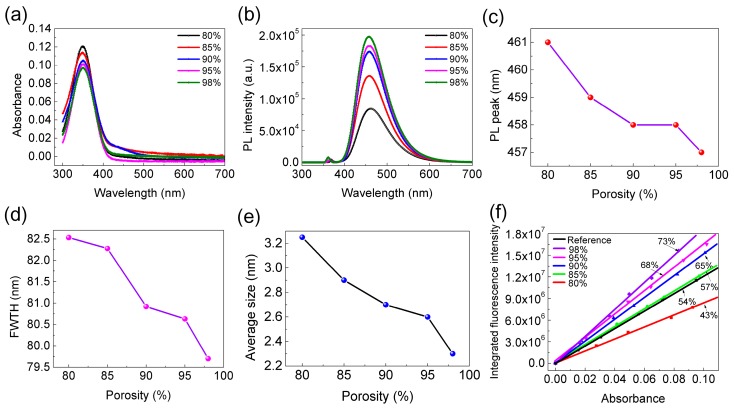
(**a**) The UV-Vis absorption spectra, (**b**) PL emission spectra, (**c**) PL peak position corresponding to (**b**) PL emission spectra, (**d**) FWHM, (**e**) average particle size, and (**f**) PL QY of N-CDs synthesized by using a microreactor with 98%, 95%, 90%, 85%, and 80% porosities of copper fibers, respectively.

In addition, we investigated the effect of different porosities of copper fibers on the optical properties of the N-CDs, as shown in Figure 3a–f. The synthesis conditions were optimized as above. Figure 3a shows that all the samples have the same and strong first characteristic absorption peak at about 350 nm. Keeping the same absorbance at 365 nm, the relationships between PL intensity and the porosity of copper fibers are shown in Figure 3b. As the porosity of copper fibers ranges from 98% to 80%, the PL intensity remarkably decreases. In addition, the PL peak position, the full width at half maximum (FWHM), the average particle size, and the PL QYs of the N-CDs are also studied, as presented in Figure 3c–f. Interestingly, the variations in PL peak position, the FWHM, and average particle size of N-CDs show a similar increase corresponding to the porosity of copper fibers ranging from 98% to 80%, which is possibly related to size effect of the N-CDs. Moreover, the PL QY of N-CDs is calculated by combining absorbance (365 nm) with PL intensity integral area, shown in Figure 3f. As the porosity of copper fibers decreases (98% to 80%), the PL QY reduces nearly two-fold. The results reveal that the microreactor with different porosities of copper fibers has a great effect on the structural and optical properties of N-CDs. Therefore, according to the above analysis, the optimum synthesis conditions for N-CDs with a maximum PL QY of 73% were a reaction temperature of 210 °C, a flow rate of 20 mL/min, an EDA dosage of 1.0 mL, a porosity of copper fibers of 98%, and a total reaction time of <6 min, shown to be more effective than most previously reported approaches (Table 1).

**Figure 4:**
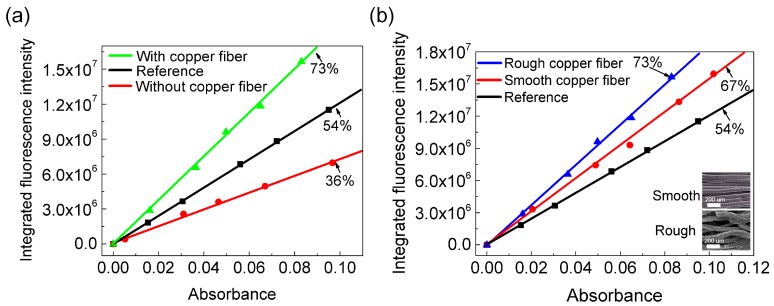
(**a**) The PL QY of N-CDs synthesized at different conditions. The black line represents the reference (QS as the standard), while the green line and red line represent the reaction region with and without copper fibers, respectively. (**b**) The PL QY of N-CDs synthesized by different surface morphologies of copper fibers. The blue and red line represent the copper fibers with a rough and smooth structure, respectively; the black line represents the reference (QS as the standard). Insets are SEM images of copper fibers with a rough and smooth structure, respectively.

Furthermore, the PL QY of the N-CDs synthesized by using the microreactor with and without copper fibers were investigated. All the synthesis conditions were the same except for with or without copper fibers. Figure 4a clearly demonstrates that the PL QY of the N-CDs is two-fold higher when the copper fibers are used. The improved PL QY is due to copper fibers that have quick heating and mass transfer properties, as well as a rapid mixing ability. As the results in Figure 2 show, the reaction temperature of the microreactor has a great effect on the properties of N-CDs. When the mixture of CA, water, and EDA was pumped, the state of solution changed from liquid to gaseous, which influenced small molecular fluorophore generation and carbon core formation [49,50,51]. Moreover, lots of small molecular fluorophores are formed because of rapid heat transfer and quick mixing, which result in N-CDs. In addition, the copper fibers reduce the gas or liquid movement and thus create a high-pressure atmosphere, which is also good for small molecular fluorophore generation and carbon core formation. Therefore, the distinction of the PL QY between with and without copper fibers is ascribed to the extent of carbon core generation and the number of small molecular fluorophores. Besides, the effects of the surface structure of copper fibers on the PL QY are also studied, as presented in Figure 4b. The PL QY of N-CDs synthesized by rough copper fibers is higher than the smooth specimens because the former has a better heat and mass transfer capacity.

### 3.3. Determining the Relationship between Copper Fibers and N-CDs

**Figure 5:**
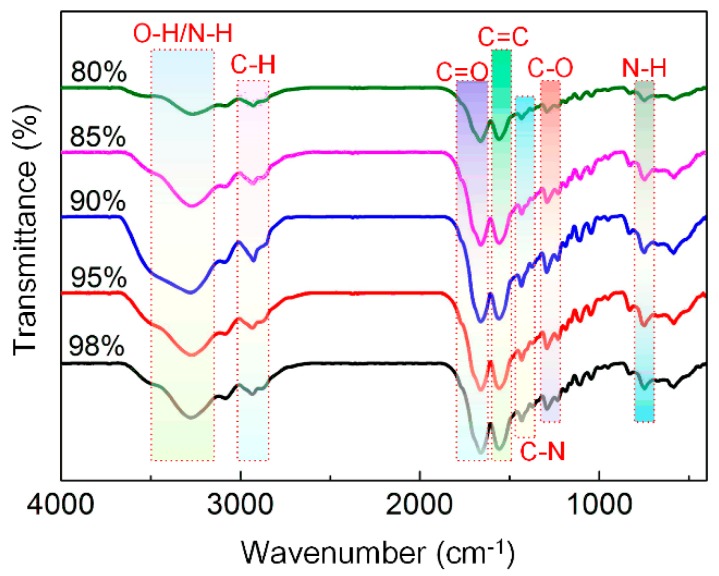
FTIR spectra of five types of N-CDs synthesized by using 98%, 95%, 90%, 85%, and 80% porosities of copper fibers, respectively.

To understand the effect of porosities of the copper fibers on the N-CDs, FTIR and XPS were performed for exploring the chemical bonding and chemical composition of the N-CDs. Figure 5 shows that the FTIR spectra of five kinds of N-CDs are similar, in addition to the relative intensities of a few bands. The stretching vibrations of O-H/N-H are located at 3410–3150 cm^−1^, indicating the generation of -OH corresponding to five kinds of N-CDs. The narrow peak located at ~2942 cm^−1^ suggests that the stretching vibrations of C-H are formed. In addition, the band at around 1240–1655 cm^−1^ is ascribed to the aromatic rings’ skeletal vibrations. To be specific, the absorption peaks at 1656 cm^−1^, 1561 cm^−1^, 1440 cm^−1^, and 1282 cm^−1^ could be attributed to C=O, C=C, C-N=, and C-O stretching, respectively. The presence of the stretching vibrations of O-H/N-H at 3400–3150 cm^−1^ and C-N= at 1440 cm^−1^ confirmed the successful surface passivation and doping of nitrogen. Here, after carefully comparing the FTIR spectra of these N-CDs, we found that the center of the absorption peak of the O-H/N-H bonds transfers from 3250 to 3300 cm^−1^ as the porosities of the copper fibers ranged from 98% to 80%. This result can be ascribe to the effect of amidogen modification on the N-CDs. Additionally, the C-H and C-O bands at approximately 2942 and 1282 cm^−1^ first increased and then decreased as the copper fibers’ porosities decreased, which may be related to some of the carbon atoms’ oxidization and carbonization.

**Figure 6:**
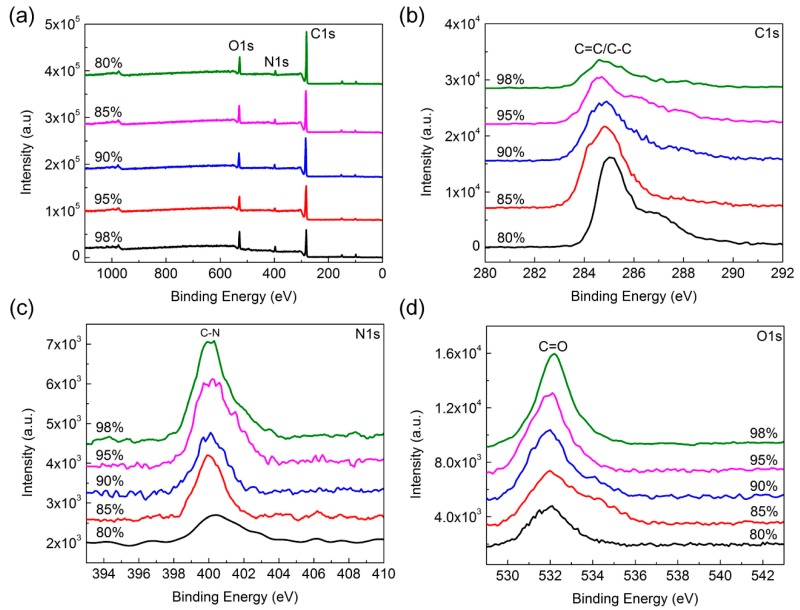
(**a**) Full-scan XPS spectra and (**b**–**d**) C1s, N1s, and O1s HR-XPS spectra of the N-CDs synthesized at different porosities of copper fibers: 98%, 95%, 90%, 85%, and 80%, respectively.

XPS measurements were applied to determine the chemical bonding and chemical composition of the N-CDs. The full-scan XPS spectra and high-resolution XPS (HR-XPS) spectra of C1s, O1s, and N1s are presented in Figure 6a–d, respectively. The full-scan XPS spectra in Figure 6a show that five types of N-CDs are composed of C, N, and O, and display three peaks of C1s at 284.6 eV, N1s at 400.7 eV, and O1s at 532.8 eV. The atomic percentages corresponding to HR-XPS spectra of C1s, N1s, and O1s are depicted in Table S1, demonstrating that the N element has been embedded in the framework of the N-CDs. Moreover, Table S1 indicates that the content of C increases as the porosities of copper fibers decrease, while the N and O contents decrease. The N-CDs with 98% porosity of copper fibers have the highest N and O content, suggesting that this is one of the reasons for the highest PL QY. In addition, a lower porosity of copper fibers enhances the carbonization degree, and higher N contents are beneficial to producing a high PL QY. The HR-XPS spectra fitting is shown in Figure S2 in the Supplemental Materials. The C1s spectrum (Figure 6b) shows one characteristic peak located between 284.6 and 285.0 eV, consistent with the C-C and C=C of the graphitic structure. The N1s XPS spectrum (Figure 6c) has a comparatively stronger signal, indicating a higher N doping level. In addition, the N1s peak is located at around 400.1 eV, suggesting that the main state of the N element is pyridinic, rather than graphitic [7]. This result reveals that a number of N was inserted in the defect area or surface of the N-CDs. Besides, the intensity of the N1s peak decreases as the copper fibers’ porosity decreases, which indicates that the N doping content decreased and is consistent with Table S1. The O1s peaks are centered at about 532 eV (Figure 6d), suggesting that oxidation occurred in the graphitic structure of the N-CDs. Moreover, the peaks of C1s and N1s gradually shift to a higher energy state, while the peak of O1s slightly shifted to a lower energy. The FTIR and XPS results are coincident, both confirming that the N element was incorporated into the N-CDs. Meanwhile, they reveal that the five types of N-CDs contain a number of surface functional groups (C-O, C-C, N-H, and O-H), implying that the samples exhibited excellent water solubility without further processing. Therefore, these N-CDs show great prospects for applications in bioscience and toxic or hazardous materials detection.

### 3.4. Detection of Hg^2+^ Ions

**Figure 7:**
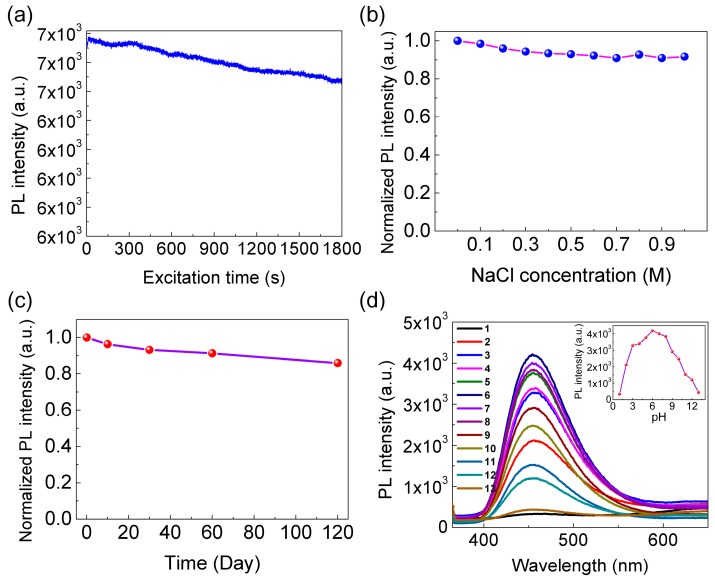
(**a**) The photostability of N-CDs under 365 nm UV light continuous excitation. (**b**) The stability of the N-CDs at different NaCl concentrations. (**c**) The stability of the N-CDs at various storage periods under ambient air. (**d**) The variation of the N-CDs PL intensity under different pH values (1~13).

The photostability of the N-CDs was studied under 365 nm UV light continuous excitation, as shown in Figure 7a. After 30 min, the PL intensity decreased by 2.19%, demonstrating that the N-CDs possess a high photostability under an ambient environment. In addition, the stability of N-CDs was further studied at various storage periods and different physical salt concentrations at room temperature, as shown in Figure 7b,c, demonstrating a high stability. These stability studies suggest that the N-CDs have good potential for practical applications. Besides, previous research has shown that the environmental pH has an important effect on the luminescence of N-CDs. Therefore, the pH-dependent fluorescence property of the N-CDs was studied, as shown in Figure 7d. The PL intensity of N-CDs varies remarkably over a wide range of pH. Starting at a low pH, the PL intensity increases by degrees to a peak value at a pH of 6.3, followed by a decrease at a higher pH. Under a strong acidic or alkaline condition, the PL was greatly weak. The possible reason for this is that the carboxyl and hydroxyl functional groups on the surface of N-CDs are prone to aggregation due to strong intermolecular hydrogen bonding between carboxyl and hydroxyl groups under an extreme pH. This result confirms that the as-synthesized N-CDs were pH-dependent.

**Figure 8:**
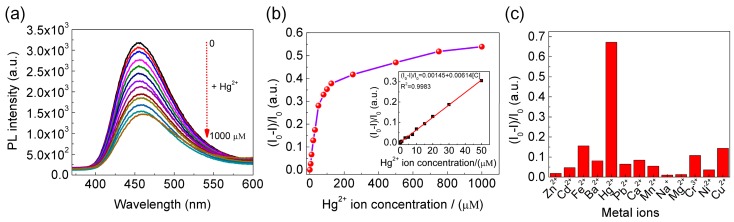
(**a**) PL emission spectra of the N-CDs upon the addition of Hg^2+^ ions with different concentrations (0~1000 μM). The lines with different colors represent different concentrations (0~1000 μM), and arrow direction represents Hg^2+^ concentrations increase. (**b**) PL intensity response of N-CDs to different concentrations of Hg^2+^ ions. The inset demonstrates the relationship between (I_0_ − I)/I and Hg^2+^ ions concentrations over the range of 0~50 μM. (**c**) The change of PL intensity at 465 nm for N-CDs with the addition of various metal ions.

To demonstrate their promising prospects for applications in biomedical and environmental sensing, we applied the N-CDs to the detection of Hg^2+^. As shown in Figure 8a, the PL intensity of N-CDs declined as the concentrations of Hg^2+^ increased, demonstrating that the N-CDs are very sensitive to Hg^2+^ ions and could be used for their detection. Figure 8b clearly exhibits that the rate of the PL intensity decay of N-CDs increased as the concentration of Hg^2+^ ions increased. The linear relationship between (I_0_ − I)/I and Hg^2+^ ions concentrations can be calculated using the Stern-Volmer equation [52,53].

I_0_ − I/I_0_ = *b* + *K*_sv_ [C] (2)

where I and I_0_ are the PL intensity of the N-CDs at 456 nm with and without Hg^2+^ ions, respectively. The *K*_sv_ is the quenching constant of Stern-Volmer, and [C] represents the Hg^2+^ ions concentration. By using Equation (2), the LOD is calculated to be 2.54 nM, which shows the detection sensitivity of N-CDs better than most previously reported values (as displayed in Table 2). In practical applications, a fluorescent probe should be highly selective as well as ultrasensitive toward the target element. To evaluate the selectivity of the N-CDs for Hg^2+^ detection, various metal ions were added to the N-CDs solution and the change of PL intensity at 456 nm was investigated. Figure 8c and Figure S3 show that the N-CDs were only sensitive to Hg^2+^, while other metal ions detected had no distinct effect. This indicates that these N-CDs are both selective and sensitive to Hg^2+^.

### 3.5. Mechanism of Fluorescence Quenching of the N-CDs

Previous reports showed that the mechanism of fluorescence quenching of the N-CDs by Hg^2+^ was related to an inner filter effect [60], aggregation induction [61], or electron transfer [62]. To help determine the mechanism behind these effects, we conducted an investigation using fluorescence lifetime and cyclic voltammetry (CV) studies.

**Figure 9:**
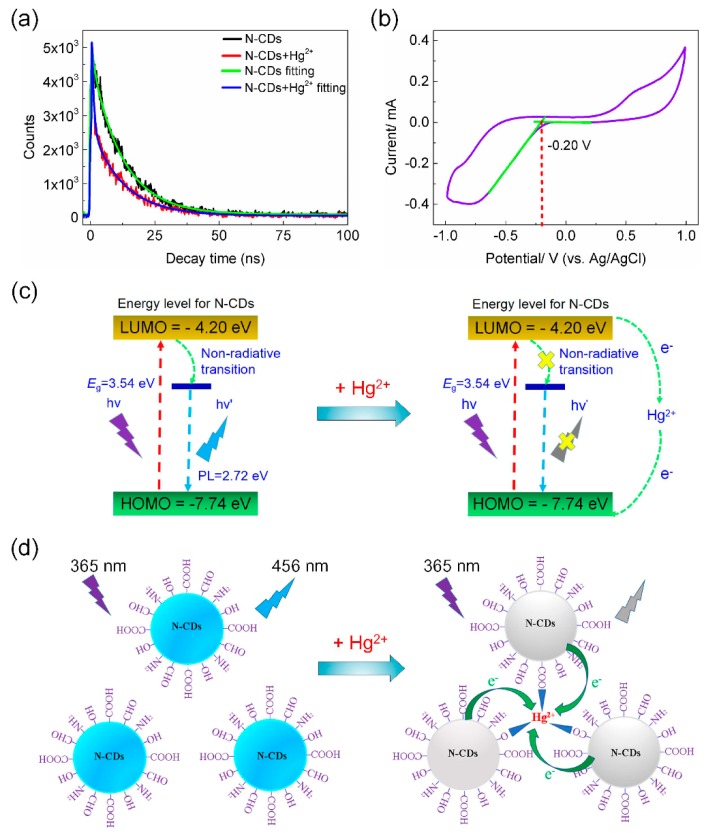
(**a**) The PL lifetime of N-CDs in the absence and presence of Hg^2+^ ions. Black and red lines are experimental data of N-CDs with and without Hg^2+^ ions, respectively; green and blue are fitting data of N-CDs with and without Hg^2+^ ions, respectively. (**b**) The CV curve of N-CDs. The green line is the linear fitting of lower CV. (**c**) Proposed energy levels related to fluorescence quenching of the N-CDs/Hg^2+^ system. (**d**) Mechanism of the fluorescence quenching of N-CDs upon the addition of Hg^2+^ ions.

Figure S4 shows that the absorption spectrum of N-CDs does not change with Hg^2+^ present, and Hg^2+^ has no absorption in the range of 250–500 nm studied, demonstrating that no inner filter effect occurred due to Hg^2+^. In addition, the TEM images and size distribution of the N-CDs (Figure S5) demonstrate that the average size of the N-CDs increased lightly from 3.25 nm to 3.42 nm after the addition of Hg^2+^, indicating no major aggregation [63]. The fluorescence lifetime of N-CDs in Figure 9a decreases from 11.71 ns to 4.24 ns in the presence of Hg^2+^, suggesting increased non-radiative recombination of the electron-hole in N-CDs due to Hg^2+^. Since Hg^2+^ has no absorption in the emission region of the N-CDs, energy transfer is not possible, leaving electron transfer from N-CDs to Hg^2+^ as a good possibility for the fluorescence quenching.

To determine the relevant energy levels of N-CDs, a standard three-electrode system was applied to test CV, which contains a platinum (Pt) sheet as the working electrode, a Pt-wire as the counter electrode, and an Ag/AgCl as the reference electrode. By utilizing CV with potential sweeping between −1.0 and 1.0 V at a scanning rate of 10 mV/s, ECL signals of the 0.10 M N-CDs are recorded and 0.10 M Bu_4_NBF_6_ (dissolved in DMF) is the supporting electrolyte. The HOMO and LUMO energy levels of N-CDs are calculated using the following equations [40,63]:

*E*_HOMO_ = −(*E*_Ox_ + 4.4) (eV) (3)

*E*_LUMO_ = −(*E*_Red_ + 4.4) (eV) (4)

*E*_HOMO_ = *E*_LUMO_ − *E*_g_ (5)

where *E*_Red_ and *E*_Ox_ represent the onset of reduction and oxidation potential for N-CDs, respectively. *E*_g_ is the HOMO/LUMO energy level gap, which can be calculated from the UV-Vis absorption spectrum. The *E*_Red_ is calculated to be −0.20 eV (Figure 9b), and the *E*_LUMO_ is determined to be −4.20 eV. The HOMO energy level could be obtained using Equation (5), which is calculated to be −7.74 eV (Figure 9c). Both the FTIR and XPS results suggest some oxygen-containing groups (e.g., -COOH, -CO, C-O-H, C-O-O, etc.) with long pair electrons in the N-CDs, which can cooperate with Hg^2+^ ions to generate complexes, resulting in electron transfer from the N-CDs to Hg^2+^ (Figure 9d). Moreover, according to the previous reports, the introduction of N doped into the graphitic structure of N-CDs could regulate the electron density of N-CDs and promote coordination between oxygen-containing functional groups on the surface of N-CDs and Hg^2+^ [26,64]. Therefore, when Hg^2+^ is present, fast electron transfer can shift from the LUMO levels of N-CDs to Hg^2+^, leading to N-CD fluorescence quenching. The different d orbital energy levels of Hg^2+^ ions compared to those of other metal ions can affect the efficiency of electron transfer and thereby result in their different response in fluorescence quenching towards N-CDs.

## 4. Conclusions

In summary, the N-CDs with a high PL QY and stability were synthesized by applying a microreactor with different porosities of copper fibers. The as-synthesized N-CDs exhibited a absorption maximum at 350 nm and PL maximum at 456 nm. By investigating the effects of reaction temperature, flow rate, EDA dosage, and copper fibers porosity on the optical properties of N-CDs, the optimal synthesis conditions for N-CDs with a high PL QY of up to 73% were found to be a reaction temperature of 210 °C, flow rate of 20 mL/min, EDA dosage of 1.0 mL, and copper fiber porosity of 98%. Compared to without copper fibers, the PL QY of N-CDs was two times higher with copper fibers. Additionally, the PL QY of N-CDs synthesized via rough copper fibers was higher than smooth copper fibers. The relationship between the copper fibers with different porosities and molecular structures of the N-CDs was studied, demonstrating that the elemental contents and surface functional groups of the N-CDs were significantly influenced by the porosity of copper fibers. The N-CDs were applied to test Hg^2+^ ions, and the result showed a good linear response in the 0~50 μM Hg^2+^ ions concentration range and the LOD of 2.54 nM. Moreover, Hg^2+^ may form coordination complexes with oxygen functional groups of N-CDs, resulting in electron transfer and a strong fluorescence quenching of N-CDs. This mechanism forms the basis of the rapid and selective detection of Hg^2+^.

## Figures and Tables

**Table 1:** Comparison of the PL QY of the CDs synthesized by different methods.

Type	Methods	Reaction Conditions	PL QY (%)	References
Bottom up	Microwave	150–300 °C, 5 h	80.6	[40]
	Ultrasonic	3 h	28.3	[41]
	Thermal combustion	170 °C, 90 min	73.2	[42]
	Hydrothermal	110 °C, 2 h	48.3	[43]
	Acid treatment	HNO_3_, 24 h	1.1–6.3	[44]
Top down	Laser ablation	20 Hz, 2 h	36	[45]
	Arc discharge	100–150 A, 60 V	2.5–6.5	[46]
	Electrochemical exfoliation	Alcohols, NaOH, 4 h	15.9	[47]
	Oxidative acid treatment	H_2_O_2_, NaNO_2_	12	[48]
	Microreactor	150–230 °C, <6 min	73	This work

**Table 2:** Comparison of the performance of the as-synthesized N-CDs with other fluorescent probes towards Hg^2+^ detection.

Fluorescence Probes	LOD (nM)	Linear Range (μM)	PL QY	References
N-CDs	230	0–25	15.7	[3]
Au/N-CDs	118	0–41.86	8.6%	[5]
N,P-CDs	1	0–0.9	53.8%	[7]
CDs	201	0–80	82.4%	[54]
N-CDs	80	0–300	38.4%	[26]
N-CDs	7.3	0.05–5	42.5%	[17]
N,S-CDs	6.5	0.01–0.25	31.8%	[55]
CDs	4.2	0–3	11.0%	[56]
CDs	10	0–5	68%	[57]
N,S-CDs	50	0.06–5	17.59	[58]
AA-CDs	5.5	0–0.05	45.1%	[59]
N-CDs	2.54	0–50	73%	This work

## Supplementary Materials

The following are available online at http://www.mdpi.com/2079-4991/8/11/900/s1, Figure S1: Photographs of the N-CDs under the UV light and N-CDs powder, Figure S2: (a–c) XPS peak differentiation-imitating analysis of C, N, and O, respectively, Figure S3: Photographs of N-CDs with the addition of different metal ions under 365 nm irradiation, Figure S4: UV-vis absorption spectra of N-CDs in the absence and presence of Hg^2+^ and Hg^2^+, Figure S5: TEM images and size distribution of N-CDs in the absence and presence of Hg^2+^, Table S1: The atomic percentages corresponding to C1s, N1s, and O1s peaks of the centre binding energies of the N-CDs.
