# Supplementary material for: Highly Photoluminescent and Stable N-Doped Carbon Dots as Nanoprobes for Hg2+ Detection
Source: Nanomaterials (Basel). 2018 Nov 2;8(11):900. doi: 10.3390/nano8110900 (PMC6265737; doi:10.3390/nano8110900)
Supplement: Supplementary file 1 [file nanomaterials-08-00900-s001.pdf]

Supporting information

Highly Photoluminescent And Stable N-Doped Carbon Dots As Nanoprobes For Hg^{2+} Detection

Longshi Rao [1, 2], Yong Tang [1], Hanguang Lu [1], Shudong Yu [1, 3], Xinrui Ding [1], Ke Xu[2, 4], Zongtao Li [1,*]

and Jin Z. Zhang[2,*]

[1] Engineering Research Centre of Green Manufacturing for Energy-Saving and New-Energy Technology, School of Mechanical and Automotive Engineering, South China University of Technology, Guangzhou 510640, China; memerls@mail.scut.edu.cn (L.R.); ytang@scut.edu.cn (Y.T.); guyuexuan1999@foxmail.com (H.L.); shudong.yu@partner.kit.edu (S.Y.); dingxr@scut.edu.cn (X.D.)

[2] Department of Chemistry and Biochemistry, University of California, Santa Cruz, CA 95064, USA; kxu26@ucsc.edu

[3] Light Technology Institute, Karlsruhe Institute of Technology (KIT), Engesserstrasse 13, 76131 Karlsruhe, Germany

[4] Department of Chemistry and Chemical Engineering, Chongqing University, Chongqing 400044, China

* Correspondence: meztli@scut.edu.cn (Z.L.); zhang@ucsc.edu (J.Z.Z.); Tel.: +86-138-2446-0886

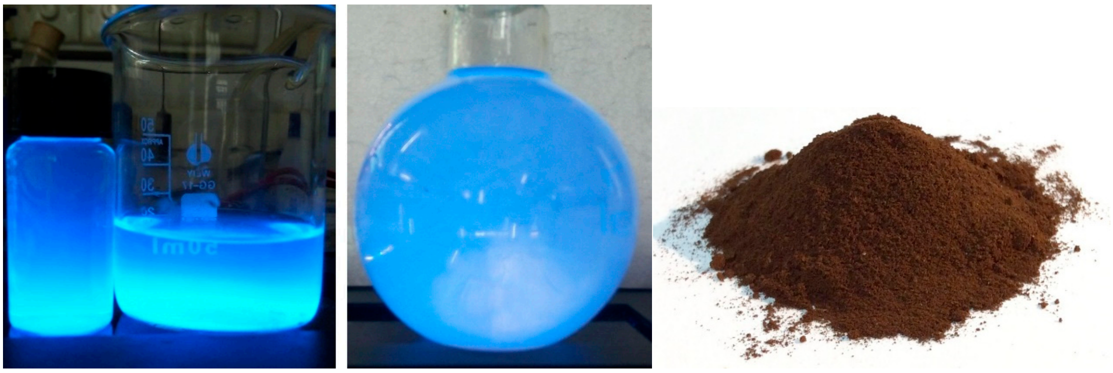

Figure S1. Photographs of the N-CDs under the UV light and N-CDs powder.

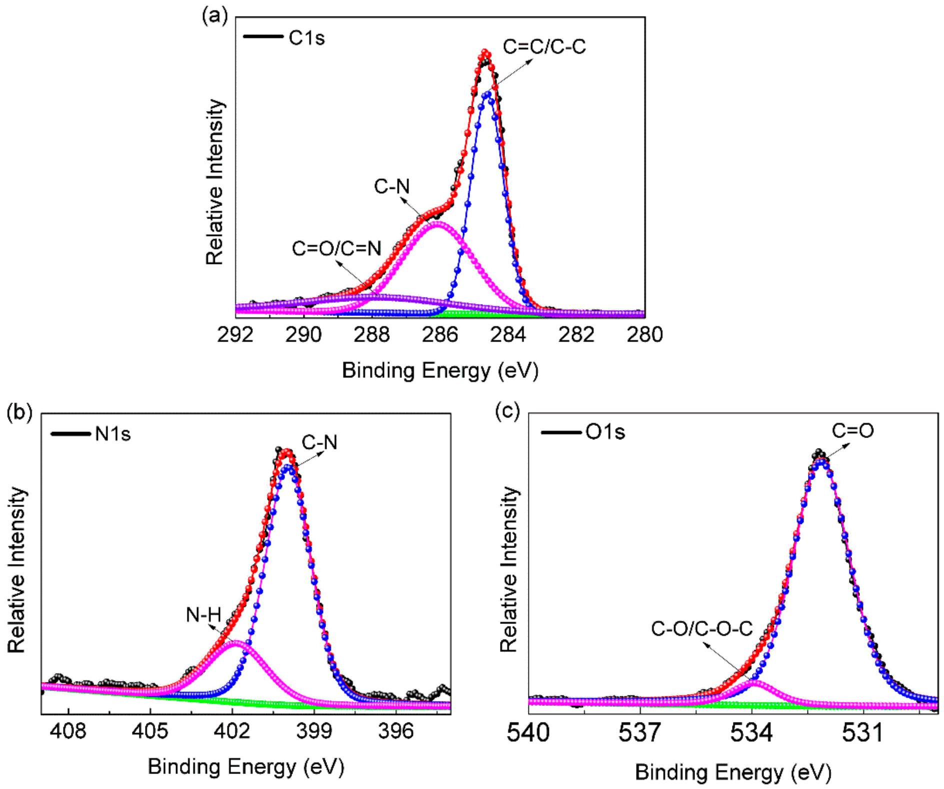

Figure S2. (a-c) XPS peak differentiation-imitating analysis of C, N, and O, respectively.

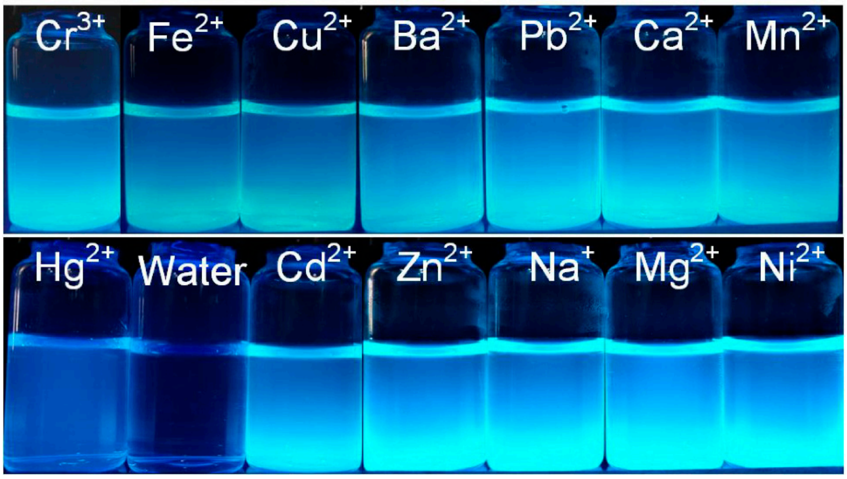

Figure S3. Photographs of N-CDs with the addition of different metal ions under 365 nm irradiation.

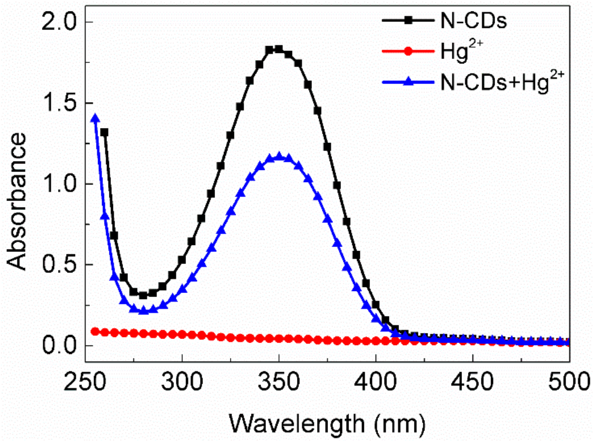

Figure S4. UV-vis absorption spectra of N-CDs in the absence and presence of Hg^{2+} and Hg^{2+}.

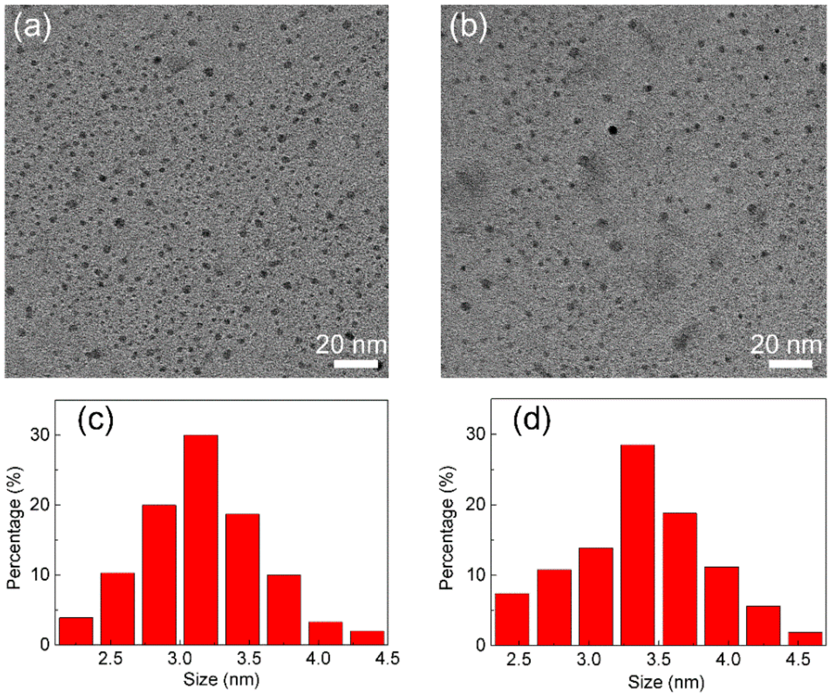

Figure S5. TEM images and size distribution of N-CDs in the absence and presence of Hg^{2+}.

Table S1. The atomic percentages corresponding to C1s, N1s, and O1s peaks of the centre binding energies of the N-CDs.

Sample	C (wt%)	N (wt%)	O (wt%)
98%	73.54	9.17	17.29
95%	78.42	8.64	12.94
90%	80.09	7.78	12.13
85%	83.78	5.35	10.87
80%	88.02	4.84	7.14
